# Lipoprotein(a) Concentration and Cardiovascular Disease in a Group of Patients With Familial Hypercholesterolemia—A Lipid Clinic Experience

**DOI:** 10.1002/clc.70125

**Published:** 2025-05-07

**Authors:** J. Kurdziel, A. Fedak, E. Kawalec, P. Miarka, A. Micek, M. Małecki, M. Walus‐Miarka

**Affiliations:** ^1^ Department of Metabolic Diseases and Diabetology University Hospital Krakow Poland; ^2^ Department of Radiology Jagiellonian University Medical College Krakow Poland; ^3^ Department of Metabolic Diseases and Diabetology Jagiellonian University Medical College Krakow Poland; ^4^ Department of Nephrology Jagiellonian University Medical College Krakow Poland; ^5^ Statistical Laboratory, Institute of Nursing and Midwifery, Jagiellonian University Medical College, Faculty of Health Sciences Krakow Poland; ^6^ Department of Medical Education Center for Innovative Medical Education, Jagiellonian University Medical College Krakow Poland

**Keywords:** familial hypercholesterolemia, intima‐media thickness, lipoprotein(a)

## Abstract

**Background:**

High Lp(a) concentrations are linked to an increased risk of cardiovascular disease (CVD). However, more evidence is needed to assess the association of Lp(a) with CVD in different vascular beds.

**Hypothesis:**

The aim was to assess the prevalence of increased Lp(a) levels and the association between Lp(a) levels and CVD in hypercholesterolemic patients.

**Methods:**

We examined 220 patients (110 women) with suspicion of FH. The mean (SD) age was 49.1 (15.02) years, LDL‐C 3.49 (1.75) mmol/L, and the median (IR) Lp(a) concentration 0.15 (0.53) g/L.

**Results:**

CVD was present in 24.5%, coronary artery disease (CAD) in 21.4% of the examined individuals. Patients with CVD and patients with CAD had higher Lp(a) levels than patients without these diseases (*p* = 0.0403 and *p* = 0.0063, respectively); however, after adjustment for age and sex, only the difference in Lp(a) concentrations between persons with and without CAD remained significant. In total, 42.3% of patients who underwent carotid ultrasound examination had carotid plaques. We did not observe differences in Lp(a) levels between patients with or without plaques or correlations between Lp(a) and carotid IMT. In total, 28.3% of patients had Lp(a) concentrations in the high (> 0.5 g/dL), and 9.9% in the moderate‐risk category (0.3–0.5 g/L). We observed an association between Lp(a) risk categories and the presence of CVD (*p* = 0.003) and CAD (*p* = 0.0004) but not with the presence of carotid plaques.

**Conclusions:**

We found a high prevalence of increased Lp(a) levels in a group of hyperlipidemic persons and strong associations of Lp(a) risk categories with CAD but not with carotid atherosclerosis.

## Graphical abstract

Average Lp(a) levels were significantly higher in FH patients with coronary artery disease compared to those without. However, no differences in Lp(a) levels were found between patients with or without carotid plaques after adjustment for sex and age.

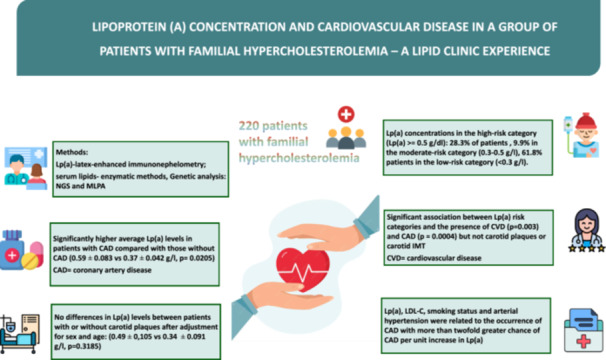

## 1 Introduction

Lipoprotein(a) [Lp(a)] is a liver‐derived lipoprotein with primarily genetically determined concentrations. There is a great interindividual variation in the serum Lp(a) concentration, with values ranging from < 0.01 to > 2 g/L in the general population. High Lp(a) levels have been linked to increased risk of coronary artery disease (CAD), and coronary atherosclerosis as well as to stroke [1, 2, 3, 4, 5]. However, there are still some gaps in knowledge concerning associations between Lp(a) and measurements of atherosclerotic changes [6, 7]. Moreover, increased Lp(a) levels were associated with an increased risk of degenerative aortic valve stenosis and heart failure [8, 9]. Increased Lp(a) levels were also associated with asymptomatic carotid artery disease in a large randomized population and carotid plaque formation in diabetic patients [10, 11]. However, more evidence is needed to assess the associations between Lp(a) and the development of CVD in different group of patients.

The aim of this study was to assess the prevalence of increased Lp(a) levels in hyperlipidemic patients treated at outpatient lipid clinics and to assess the relationships between Lp(a) levels and CVD in this group of patients.

## 2 Materials and Methods

We examined 220 consecutive patients (110 women) referred to a lipid outpatient clinic with suspicion of familial hypercholesterolemia (FH). The data of each patient, including past history, family history, smoking habits, and drug use, were collected via a standardized questionnaire. Anthropometric measurements included weight, height, waist and hip circumference, and blood pressure. Fasting blood samples were collected for determination of the concentrations of serum lipids, glucose, creatinine, HbA1c, and Lp(a). Serum lipids were determined by enzymatic methods, LDL‐cholesterol (LDL‐C) concentrations were determined by direct measurement, and Lp(a) levels were determined using latex‐enhanced immunonephelometry, with a normal reference value < 0.3 g/L. Patients with Lp(a) values between 0.3 and 0.5 g/L were classified as having moderate cardiovascular risk, patients with values above 0.5 g/L were classified as high risk, and patients with values above 1.8 g/L were classified as very high risk [5, 12]. Cardiovascular disease (CVD) was diagnosed if the patient had a past history of myocardial infarction, stent placement, CABG, coronary arterial vessel obstruction above 50%, stroke, or transient ischemic attack. CAD was defined as a past history of myocardial infarction, stent placement, CABG, or arterial vessel obstruction above 50%. Arterial hypertension was defined as arterial pressure above 130/80 mmHg or treated hypertension. FH was diagnosed using the Simon Broome Register criteria [13]. Genetic analysis was performed using NGS and MLPA [14, 15]. Carotid ultrasound was performed by an experienced radiologist. The intima‐media thickness (IMT) of the proximal and distal common carotid arteries as well as the bifurcation of both the left and right carotid arteries were measured. Carotid plaque was defined as a local enlargement of the IMT of more than 50% of the surrounding IMT or an IMT above 1.5 mm and was documented as present or absent.

Statistical analysis included calculations of means (*x*) and standard deviations (SD) for continuous normally distributed variables or medians (Me) and interquartile ranges (IR) for variables that were not normally distributed. Univariate logistic regression analysis and backward stepwise multivariable logistic regression analysis including Lp(a), BMI, LDL‐C, smoking status, diabetes status, and arterial hypertension status (after adjustment for age and sex) were performed to assess which factors are associated with the occurrence of CAD.

The study was performed in accordance with the principles of the Declaration of Helsinki, and all patients provided written informed consent before inclusion. Institutional review board approval was obtained from Bioethics Committee of the Jagiellonian University No. 1072.6120.133.2022.

## 3 Results

We examined 220 consecutive hyperlipidemic patients (110 women), and the mean age of the patients was 49.1 ± 15.0 years. The characteristics of the patients in the studied group are presented in Table 1. The median (interquartile range, IR) Lp(a) concentration in the examined group was 0.15 (0.53) g/L, the mean (SD) LDL‐C level was 3.49 (1.75) mmol/L, and the mean BMI was 26.44 (4.55) kg/m^2^. CVDs were present in 24.5%, CAD in 21.4%, type 2 diabetes in 17.3%, and arterial hypertension in 40.0% of patients. Genetic analysis was performed in 81 patients and FH was confirmed in 50 patients (61.7%) of the examined individuals. A total of 30.9% of patients were current smokers. Most patients (84.5%) were treated with statins. The percentage of patients treated with statins was greater in persons with CVD than in those without CVD (*p* = 0.035) and in patients with CAD than in those without CAD, although this difference did not reach statistical significance.

**Table 1:** Characteristics of the studied group.

Parameters	Total		
	*N* = 220		
	*n*	*x*/Me	SD/IR
Age [yrs]	220	49.1	15.02
TC [mmol/L]	219	5.61	1.922
HDL‐C [mmol/L]	219	1.46	0.478
LDL‐C [mmol/L]	220	3.49	1.75
TG [mmol/L]^a^	220	1.33	1.26
Lp(a) [g/L]^a^	220	0.15	0.53
Lp(a) [g/L]	220	0.41	0.55
Glucose [mmol/L]^a^	177	5.28	0.83
Creatinine [mg/L]^a^	209	74.0	20.5
BMI [kg/m^2^]	220	26.44	4.558
Alat [U/L]^a^	217	29.0	21.0
SBP [mmHg]	220	129.3	12.46
DBP [mmHg]	220	79.5	8.57
HbA1c [%]^a^	54	5.90	1.00

Parameters	*N* = 220	
	*n*	%
Sex		
Men	110	50.0
Women	110	50.0
CVD (yes)	54	24.5
CAD (yes)	47	21.4
Obesity (BMI > 30 kg/m^2^)	46	20.9
Smoking habit (yes)	68	30.9
Statin use (yes)	186	84.5
Statin use CVD (+) pts	52	94.4*
Statin use CVD (−) pts	135	81.3
Statin use CAD (+) pts	44	93.6
Statin use CAD (−) pts	142	82.1
Diabetes (yes)	38	17.3
Hypertension (yes)	88	40.0
FH (yes)	81	36.8

^a^Median. IR—interquartile range.

* *p* = 0.035 CVD+ versus CVD−.

There were no differences in Lp(a) levels between men and women or between patients with and without diabetes or hypertension. A comparison of Lp(a) levels according to the presence of vascular disease revealed that the median (IQR) Lp(a) concentration in patients with CVD was significantly greater than that in patients without CVD (0.33 (0.69) g/L vs. 0.13 (0.34) g/L, *p* = 0.0403) and in patients with CAD than in patients without CAD (0.52 (0.92) g/L vs. 0.13 (0.34) g/L, *p* = 0.0063).

Table 2 shows the mean values of the examined variables after adjustment for age and sex according to the presence of CVD. The differences in Lp(a) concentrations between patients with and without CVD were no longer significant. However, significant differences in Lp(a) levels persisted between patients with and without CAD: *x* (SE) 0.59 (0.083) and 0.37 (0.042) g/L, respectively, *p* = 0.0205 (Table 2). After standardization for age and sex, the mean total cholesterol and LDL‐C levels were significantly lower in patients with CVD and CAD than in persons free of these diseases. LDL‐C (*x*, SE) in patients with versus without CVD was 3.01 (0.241) and 3.65 (0.133) mmol/L, respectively (*p* = 0.0254), while in persons with versus without CAD, it was 2.95 (0.258) versus 3.64 (0.129) mmol/L (*p* = 0.0203).

**Table 2:** The mean values of the examined variables in patients with and without cardiovascular disease (CVD), coronary artery disease (CAD), and carotid plaques and after adjustment for age and sex.

Parameters	Presence of the disease		Absence of disease		*p*
	*n*	*x* (SE)	*n*	*x* (SE)	
Cardiovascular diseases (CVD) *N* = 220					
TC [mmol/L]	54	4.99 (0.262)	165	5.82 (0.144)	**0.0074**
HDL‐C [mmol/L]	54	1.38 (0.063)	165	1.49 (0.035)	0.1448
LDL‐C [mmol/L]	54	3.01 (0.241)	166	3.65 (0.133)	**0.0254**
TG [mmol/L]	54	1.92 (0.276)	166	1.98 (0.152)	0.86
Lp(a) [g/L]	54	0.53 (0.078)	166	0.38 (0.043)	0.0855
Glucose [mmol/L]	44	5.68 (0.185)	133	5.46 (0.105)	0.3216
Creatinine [mg/L]	53	71.48 (3.131)	156	78.45 (1.759)	0.0598
BMI [kg/m^2^]	54	26.7 (0.605)	166	26.36 (0.333)	0.6324
Alat [U/L]	52	37.38 (3.019)	165	34.59 (1.633)	0.4293
SBP [mmHg]	54	130.4 (1.698)	166	128.9 (0.934)	0.4374
DBP [mmHg]	54	78.8 (1.221)	166	79.7 (0.672)	0.5331
HbA1c [%]	19	6.91 (0.242)	35	6.04 (0.158)	**0.0042**
Coronary artery disease (CAD) *N* = 220					
TC [mmol/L]	47	4.95 (0.28)	172	5.8 (0.141)	**0.0092**
HDL‐C [mmol/L]	47	1.37 (0.068)	172	1.48 (0.034)	0.1434
LDL‐C [mmol/L]	47	2.95 (0.258)	173	3.64 (0.129)	**0.0203**
TG [mmol/L]	47	2.04 (0.296)	173	1.94 (0.148)	0.7597
Lp(a) [g/L]	47	0.59 (0.083)	173	0.37 (0.042)	**0.0205**
Glucose [mmol/L]	37	5.74 (0.201)	140	5.46 (0.101)	0.2181
Creatinine [mg/L]	46	69.32 (3.336)	163	78.76 (1.704)	**0.0145**
BMI [kg/m^2^]	47	26.53 (0.648)	173	26.42 (0.325)	0.8762
Alat [U/L]	45	34 (3.243)	172	35.59 (1.596)	0.6684
SBP [mmHg]	47	131.2 (1.814)	173	128.8 (0.91)	0.2436
DBP [mmHg]	47	78.7 (1.306)	173	79.7 (0.656)	0.5187
HbA1c [%]	18	6.7 (0.246)	36	6.04 (0.155)	**0.0024**
Carotid plaques (*N* = 77)					
TC [mmol/L]	33	5.34 (0.39)	44	5.9 (0.34)	0.3237
HDL‐C [mmol/L]	33	1.46 (0.084)	44	1.63 (0.073)	0.1652
LDL‐C [mmol/L]	33	3.4 (0.359)	44	3.72 (0.313)	0.5368
TG [mmol/L]	33	1.64 (0.168)	44	1.34 (0.146)	0.2142
Lp(a) [g/L]	33	0.49 (0.105)	44	0.34 (0.091)	0.3185
Glucose [mmol/L]	31	5.51 (0.19)	32	5.25 (0.198)	0.3808
Creatinine [mg/L]	31	72.24 (3.979)	41	80.44 (3.463)	0.1632
BMI [kg/m^2^]	33	25.24 (0.751)	44	25.96 (0.654)	0.5136
Alat [U/L]	33	38.15 (4.021)	43	32.14 (3.539)	0.31
SBP [mmHg]	33	129.9 (2.366)	44	130.9 (2.062)	0.7641
DBP [mmHg]	33	80.3 (1.735)	44	80.4 (1.512)	0.9725
HbA1c [%]	12	6.31 (0.223)	5	5.76 (0.293)	0.2111

*Note:* Bold values indicate statistically significanct (*p* < 0.05).

Carotid artery ultrasonography was performed for 77 patients, 42.3% of whom had carotid plaques. We did not observe differences in Lp(a) levels between patients with or without carotid plaques after adjustment for sex and age: *x* (SE) was 0.49 (0.105) g/L versus 0.34 (0.091) g/L, *p* = 0.3185, respectively (Table 2).

A total of 28.3% of patients had Lp(a) concentrations in the high‐risk category (Lp(a) ≥ 0.5 g/dL), 9.9% in the moderate‐risk category (0.3–0.5 g/L), and the remaining patients in the low‐risk category (< 0.3 g/L). As expected, we observed a significant association between Lp(a) risk categories and the presence of CVD (*p* = 0.003) and the presence of CAD (*p* = 0.0004) (Table 5). There was also a significant difference in the presence of CVD between patients with Lp(a) levels above (34.12%) and below (18.12%) 0.3 g/L (*p* = 0.0068). Interestingly, we did not observe any associations between Lp(a) risk categories and the presence of carotid plaques or carotid IMT (Table 3).

**Table 3:** Prevalence of CVD, CAD, and carotid plaques according to Lp(a) serum concentration categories.

Lp(a) risk category	High risk			Moderate risk			Low risk			*p*
	Lp(a) ≥ 0.5 g/L			0.3 ≤ Lp(a) < 0.5 g/L			Lp(a) < 0.3 g/L			
	*N* = 62			*N* = 21			*N* = 137			
	28.2%			9.5%			62.3%			
	*n*	%		*n*	%		*n*	%		
CVD present	25	40.3		4	19.0		25	18.2		0.0030
CVD absent	37	59.7		17	81.0		112	81.8		
CAD present	24	38.7		4	19.0		19	13.9		0.0004
CAD absent	38	61.3		17	81.0		118	86.1		

Carotid usg, *N* = 77	*N* = 23			*N* = 8			*N* = 46			*p*
	*n*	%		*n*	%		*n*	%		
Carotid plaques present	13	56.5		3	37.5		17	37.0		0.2863
Carotid plaques absent	10	43.5		5	62.5		29	63.0		
IMT R (*n*, Me, IR)	17	0.80	0.40	7	0.80	0.20	40	0.80	0.45	0.7347
IMT L (*n*, Me, IR)	17	0.90	0.50	7	0.80	0.20	40	0.76	0.49	0.5271

IMT L = thickness of LCCA [mm]

IMT R = thickness of RCCA [mm]

Univariate logistic regression analysis of variables such as Lp(a), BMI, LDL‐C, smoking status, diabetes status, and arterial hypertension after adjustment for age and sex revealed that in our group of FH patients, Lp(a), LDL‐C, smoking status, and arterial hypertension were significantly and independently associated with CAD but not BMI or diabetes mellitus (Table 4).

**Table 4:** Odds ratios and 95% confidence intervals for association between examined variables and occurrence of CAD, after adjustment for age and sex—univariate logistic regression analysis.

Characteristics		95% confidence intervals		
	OR	Low limit	Upper limit	*p*
Lp(a) [g/L]	1.91	1.07	3.41	0.028
BMI [kg/m^2^]	1.01	0.93	1.10	0.761
LDL‐C [mmol/L]	0.71	0.54	0.93	0.012
Diabetes	1.62	0.70	3.77	0.261
Smoking	4.30	2.03	9.11	< 0.001
Arterial hypertension	9.76	3.76	25.31	< 0.001

A backward stepwise multivariable logistic regression analysis including Lp(a), BMI, LDL‐C, smoking status, diabetes status, and arterial hypertension after adjustment for age and sex showed that Lp(a), LDL‐C, smoking status, and arterial hypertension were related to the occurrence of CAD. An increase in the Lp(a) concentration of 1 g/L increased the chance of developing CAD more than twofold (OR = 2.25; *p* = 0.022), and smokers had a nearly fourfold greater chance of developing CAD than nonsmokers (OR = 3.87; *p* < 0.001). Arterial hypertension also increases the chance of developing CAD, although the result for hypertension should be treated with caution, as the confidence interval for the odds ratio is wide due to the small number of patients, which means that the estimate is not precise (Table 5).

**Table 5:** Odds ratios and 95% confidence intervals for association between examined variables and occurrence of CAD, after adjustment for age and sex multivariate regression logistic analysis reduced model.

Characteristic		95% confidence interval		
	OR	Low limit	Upper limit	*p*
Lp(a) [g/L]	2.25	1.13	4.50	0.022
LDL‐C [mmol/L]	0.69	0.52	0.92	0.012
Smoking	3.87	1.73	8.69	0.001
Arterial hypertension	11.85	4.76	29.51	< 0.001

Univariate logistic regression analysis examining the association of risk factors with the presence of carotid plaques, adjusted for age and sex, revealed that only smoking habit was significantly associated with carotid plaques (OR = 4,96; *p* = 0,019); however, the interpretation is difficult due to the small sample size.

There were no differences in Lp(a) concentration between patients with and without a family history of CVD. In persons with a positive family history of CVD (*n* = 69), the median (interquartile range IQR) Lp(a) concentration was 0.15 (0.32) g/L, while in persons with a negative family history, it was 0.16 (0.63) g/L (*p *= ns). Analysis of positive family history of CVD according to Lp(a) concentration risk categories did not reveal any significant associations.

## 4 Discussion

Among our group of FH patients treated in lipid outpatient clinics in the southeastern part of Poland, 38.29% had Lp(a) levels above 0.3 g/L, and 28.3% had Lp(a) levels above 0.5 g/L. This percentage is > 10%–20% observed in the general population [1]. This finding might further confirm that patients with FH are characterized by higher levels of Lp(a) than the general population [16, 17]. The results of Lp(a) determination data from Polish centers in patients with CVD showed lower values of Lp(a) than we observed in our patients. In the study of Zabrze Lp(a) registry, including patients with very high cardiovascular risk, prevalence of Lp(a) above 0.3 g/L was 27% [18]. Very high levels of Lp(a) above 0.5 g/L were observed in 28.3% in our group of FH patients, while in Zabrze cohort in 20% of patients, in tertiary cardiovascular center from Kraków in 24.95% and in 13.5% of patients of outpatient cardiology clinic [18, 19, 20]. We observed, as expected, significantly greater values of serum Lp(a) in patients with CAD than in patients free of these diseases [1, 2, 3, 4, 5]. On the other hand, LDL‐C levels were lower in CAD patients than in persons free of CAD, in line with observations by Sun et al. [21] who did not find differences in LDL‐C levels between patients with and without CAD due to intensive statin treatment in CAD patients. The association between cardiovascular and CAD and Lp(a) levels in our study was confirmed by a significant increase in the incidence of CVD and CAD across Lp(a) risk categories. These observations are in agreement with the literature, indicating the strong association of Lp(a) levels with CAD and myocardial infarction [2, 3, 4, 17, 22]. Moreover, backward stepwise multivariable logistic regression analysis confirmed the independent relationship between Lp(a) and CAD. We found that an increase in Lp(a) concentration of 1 g/L increases the chance of developing CAD more than twofold, which indicates the need to introduce effective Lp(a)‐lowering treatment to prevent CAD development. It should be stressed that Lp(a) was an independent CAD risk factor despite low LDL‐C concentrations during intensive lipid‐lowering treatment. Interestingly, we did not observe any associations between Lp(a) levels and the presence of atherosclerotic plaques in carotid arteries or carotid artery IMT. Previous studies describing associations between Lp(a) and atherosclerosis in different vascular beds are controversial. Associations between Lp(a) concentrations and carotid atherosclerosis were confirmed in asymptomatic persons, patients with type 2 diabetes, and patients with metabolic syndrome [10, 23, 24, 25]. Franca and colleagues showed that Lp(a) levels above 0.3 g/L contribute significantly to the development of carotid plaques in apparently healthy participants [26]. However, in the study of Klein and colleagues, Lp(a) levels predicted differently carotid plaque area, stenosis, and occlusion; namely, Lp(a) was a significant independent predictor of baseline stenosis and occlusion but not of carotid plaque [27]. On the other hand, our observation of a lack of association between Lp(a) and carotid atherosclerosis was confirmed by data obtained in FH patients [21, 28, 29]. Sun and colleagues observed that Lp(a) level was associated with the presence and severity of CAD but not with carotid atherosclerosis in 148 patients with heterozygous FH, what is in line with our data [21]. In a cohort of 263 male participants, Ooi and colleagues reported that Lp(a) concentration was independently associated with coronary stenosis, but no significant associations between Lp(a) concentration and carotid artery plaque were detected [29]. Convincing data confirming our results came from a prospective study by Raitakari and colleagues, who reported that elevated Lp(a) levels in young people are a risk factor for adult CVD but not for increased IMT [30]. These results are in line with our findings that serum Lp(a) concentrations are not associated with carotid atherosclerosis, as measured by IMT and carotid plaques. In the study of Cao and colleagues, which included 151 patients with genotypically confirmed FH, Lp(a) levels differed only between patients with and without coronary atherosclerotic lesions but not between patients according to the presence of peripheral and carotid artery disease. In this study, multivariate regression analysis showed that Lp(a) was independently associated with CAD only [31]. In line with these results, Deniz and colleagues found in FH patients with subclinical atherosclerosis, that Lp(a) levels ≥ 0.3 g/L predicted coronary atherosclerosis, while diabetes and low Apo A‐I/Apo B ratios predicted carotid atherosclerosis, and smoking predicted femoral atherosclerosis [32]. Interestingly, recently published data by de Boer and colleagues reported that during a 20‐year follow‐up period prospective observation higher LP(a) concentrations contributed significantly to progression of carotid IMT in FH children, in opposite to our finding, while no significant association was found between Lp(a) and IMT during follow‐up in healthy siblings of FH children [33, 34].

Some recent reviews concerning the association of Lp(a) and carotid atherosclerosis indicate that the association of Lp(a) and atherosclerotic CVD is higher for coronary than for peripheral artery disease [7, 35, 36]. Garagoli and colleagues concluded that almost all analyzed studies showed a positive association between elevated Lp(a) levels and carotid vulnerable plaque features, which were not assessed in our study [7]. Also, Dalla Vestra stated that there is a well‐established and strong link between high Lp(a) concentration and coronary heart disease, but the evidence regarding peripheral artery disease and carotid atherosclerosis is not as conclusive and suggested a more important role of Lp(a) in the progression and complication of carotid plaque than in its genesis [35]. Steffen and colleagues suggested that race could be a modifying variable in Lp(a)‐related risk of carotid plaque, and Lp(a) levels may have greater influence on plaque burden in Whites than in Black individuals [37].

Lp(a) has proatherogenic, proinflammatory and prothrombotic properties [38, 39], so there is a possibility that Lp(a) has different effects on the mechanisms of atherothrombotic and atherosclerotic vascular changes. van der Valk and colleagues demonstrated that Lp(a), through its proinflammatory oxidized phospholipid content, induces monocyte trafficking to the arterial wall and mediates proinflammatory responses [38]. A study by van Dam‐Nolen and colleagues in 182 patients with symptomatic carotid stenosis showed that higher Lp(a) levels were associated with the degree of arterial stenosis and features of vulnerable plaques, such as the presence of lipid‐rich necrotic cores, thin or ruptured fibrous caps, and intraplaque hemorrhage [23]. The authors hypothesized that Lp(a) has a marginal or no influence on the size of atherosclerotic plaques but affects plaque composition [23]. Additionally, Tian and colleagues reported that higher Lp(a) levels were significantly associated with unstable carotid plaques in patients with ischemic stroke [40]. Achim and colleagues, in a recent review, discussed the heterogeneity of atherosclerotic impairment in different arterial districts [41]. The mechanisms underlying the development of atherosclerotic changes in different arterial districts and the role of Lp(a) in their development are still not well understood [42]. In our group of FH patients obesity was observed in 21% and diabetes in 17% of examined persons, however, we did not observe any association between CAD and BMI or diabetes. In opposite to our finding data from the Hellenic Familial Hypercholesterolemia Registry (HELLAS‐FH), including 1655 FH persons found that obesity was independently associated with increased prevalence of CAD in this population [43]. On the other hand, Jansen and colleagues reported that there was no significant association between BMI and CVD and cardiovascular mortality, after standardization by other risk factors in 2400 Dutch FH patients [44]. Also meta‐analysis of cardiovascular risk factors in FH patients showed associations of BMI and CVD [45], although based only on three studies, analyzing these associations. Summing up, it seems that the mechanisms of association between BMI and CVD in FH patients require further elucidation.

Many studies confirm the association between CVD and diabetes in FH patients, in opposite to our study [44, 45, 46]. In the study of Spanish Arteriosclerosis Society Dyslipidemia Registry, HeFH patients with type 2 diabetes mellitus have a higher rate of CVD and a less favorable lipid profile than FH patients without diabetes [46]. Also meta‐analysis of CVD risk factors in FH patients and study of Jansen and colleagues showed that diabetes was associated with CVD, however, based on meta‐analysis data, the authors underline the different attributable risks of examined factors. This observation might help to explain the lack of association between CVD and diabetes in our group.

Elevated Lp(a) levels are strong predictors of CVD/CAD and are genetically determined; however, surprisingly, there were no differences in Lp(a) concentration in persons with a positive family history of CVD in comparison to persons free of CVD in family members. We also did not find associations of Lp(a) level risk category with a positive family history of CVD in our group of patients. On the other hand, another study by Pederiva and colleagues of 563 FH children and adolescents revealed that subjects with Lp(a) > 0.3 g/L more frequently reported a positive family history of CVD than did subjects with Lp(a) ≤ 0.3 g/L [47].

The limitations of our study include its cross‐sectional design and relatively small sample size, so the association between carotid atherosclerosis and Lp(a) needs further robust evidence from prospective studies with larger numbers of patients. Additionally, the use of a nephelometric assay for the determination of Lp(a) did not allow for evaluating the different apoprotein(a) [apo(a)] isoforms; however, there are data indicating that the Lp(a) serum level is the main CAD risk indicator, while isoform size does not significantly contribute to risk [22].

In conclusion, in this study, we found a high prevalence of increased Lp(a) levels and, as expected, strong associations with CAD in patients with hyperlipidemias, which indicates the need for efficient lowering of Lp(a) levels to decrease coronary risk. The association of Lp(a) levels with CAD and the lack of association with carotid IMT suggest that Lp(a) has different effects on arterial walls in different vascular beds and needs to be further investigated.

Our results are in line with data indicating that a high concentration of Lp(a) is a potential novel treatment target since there is evidence that it contributes to CVD events. The questions of the magnitude of Lp(a) reduction required to produce clinical benefit are under discussion, and current investigational treatments directed toward Lp(a) are promising methods for reducing residual risk [48, 49].

## Author Contributions

M. Walus‐Miarka designed and directed the project and wrote the manuscript. J. Kurdziel was involved in planning the work and worked on the manuscript. E. Kawalec and A. Micek performed the analytic calculations. A. Fedak performed the experiments. P. Miarka was involved in manuscript edition. M. Walus‐Miarka and all authors contributed to the final version of the manuscript.

## Conflicts of Interest

The authors declare no conflicts of interest.

## Data Availability

The data that support the findings of this study are available upon request from the corresponding author, M.W.M.
